# The Recruitment and Immune Suppression Mechanisms of Myeloid‐Derived Suppressor Cells and Their Impact on Bone Metastatic Cancer

**DOI:** 10.1002/cnr2.70044

**Published:** 2025-02-13

**Authors:** Chengyuan Li, Yucheng Xue, Eloy Yinwang, Zhaoming Ye

**Affiliations:** ^1^ Department of Orthopedic Surgery, the Second Affiliated Hospital Zhejiang University School of Medicine Hangzhou China; ^2^ Orthopedics Research Institute of Zhejiang University Hangzhou China; ^3^ Key Laboratory of Motor System Disease Research and Precision Therapy of Zhejiang Province Hangzhou China

**Keywords:** bone metastasis, immune suppression, myeloid‐derived suppressor cells

## Abstract

**Background:**

MDSCs are immature neutrophils and monocytes with immunosuppressive potentials, involving mononuclear MDSCs (M‐MDSCs) and polymorphonuclear MDSCs (PMN‐MDSCs).

**Recent Findings:**

They are significant components of the tumor microenvironment (TME). Besides, recent studies also verified that MDSCs also facilitated the progression of bone metastasis by regulating the network of cytokines and the function of immune cells.

**Conclusion:**

It is necessary to summarize the mechanisms of MDSC recruitment and immunosuppression, and their impact on bone metastasis.

## 1 Introduction

Myeloid‐derived suppressor cells (MDSCs) were first reported in the late 1970s, and the studies revealed their potential immune suppression function [1]. At the outset, these immature myeloid cells were depicted as null cells, veto cells, and natural suppressor (NS) cells due to the lack of traditional membrane markers [2, 3]. Subsequent studies identified some of the membrane markers involving glutathione reductase (GR1), CD34, or CD11b [3, 4, 5]. Following research revealed that the cells with these kinds of phenotypes in bone marrow presented immune suppression function [6]. Based on this phenomenon, they were also identified as immature myeloid cells (iMC) [1]. Besides, published research also used varied nomenclature, including N2 neutrophils, tumor‐associated neutrophils (TANs), and tumor‐associated macrophages (TAMs) [7]. In 2007, the forum identified these cells as MDSCs for their phenotype and functions [1, 8].

There are two major types of MDSCs, including polymorphonuclear MDSCs (PMN‐MDSCs) and mononuclear MDSCs (M‐MDSCs), which are like neutrophils and monocytes, respectively. For a long time, human M‐MDSCs and PMN‐MDSCs were described as CD11b^+^CD15^−^HLA‐DR^low^ and CD11b^+^CD14^−^CD15^+^/CD66b^+^, while mice PMN‐MDSCs and M‐MDSCs are marked as CD11b^+^Ly6G^+^Ly6C^−^ and CD11b^+^Ly6G^−^Ly6C^+^ [9, 10, 11]. However, these markers were insufficient to distinguish the MDSCs and other mature myeloid cells. The way to define MDSCs through their immunosuppression functions was still inadequate. In recent years, different kinds of novel markers and quantitative methods have been used to identify the MDSCs. For instance, Lectin‐type oxidized LDL receptor‐1 (LOX‐1) has been used to refine the identification of PMN‐MDSCs [12], and LOX‐1^+^ PMN‐MDSCs. The density was also used as a novel measure to distinguish PMN‐MDSCs and neutrophils since the density of PMN‐MDSCs is much lower than that of neutrophils [13]. Advanced quantitative technologies, such as single‐cell RNA sequencing (scRNA‐seq) and transposase‐accessible chromatin using sequencing (ATAC‐seq), have been applied to identify the MDSCs [10]. In recent years, the mechanisms of MDSCs recruitment and immunosuppression are always the key points in MDSCs studies. In previous studies, chemokines and hypoxia microenvironment are the major causes of MDSCs migration. As for the immune suppression function, some scholars insist that this is not the inherent characteristic of MDSCs since it is induced by inflammatory cytokines in TME [14]. Some studies on chronic inflammatory conditions supported this viewpoint [15, 16]. These findings meant that we should focus on the small molecules associated with MDSCs differentiation and immunosuppression in TME. In following contents, we will review the representative and latest viewpoints and present some possible treatment methods.

Bone is a very common site for metastasis, especially in prostate cancer and breast cancer [17, 18, 19, 20]. Bone metastases always cause a poor clinical prognosis and decrease the life expectancy of patients. The progression of bone tumors is highly correlated to the bone microenvironment [21]. Bone microenvironment provides an appropriate soil for the progression of bone metastasis tumors [20, 22, 23, 24, 25]. The components of the bone microenvironment include bone marrow, extracellular matrix, bone cells (osteoclasts, osteoblasts, osteocytes, and fibroblasts) [21, 26], and MDSCs [27]. Further, targeting the microenvironment can guide some novel approaches in the clinical treatment of bone tumors [28].

In this current review, we analyzed the recruitment mechanisms of MDSCs and indicated how MDSCs induce the anergy of other immune cells in TME, such as T cells, dendritic cells, natural killer cells, and macrophages. Meanwhile, we summarized related signal pathways and demonstrated their relationship with the progression of bone metastatic tumors. Since there are few articles focusing on the MDSCs in bone metastatic tumors, we decided to introduce the roles of MDSCs in other cancers, showing some potential directions for future studies. Based on this article, future scholars may discover other novel characteristics of MDSCs and design new clinical therapy strategies for bone metastasis cancers.

## 2 MDSCs Recruitment to TME

**FIGURE 1:**
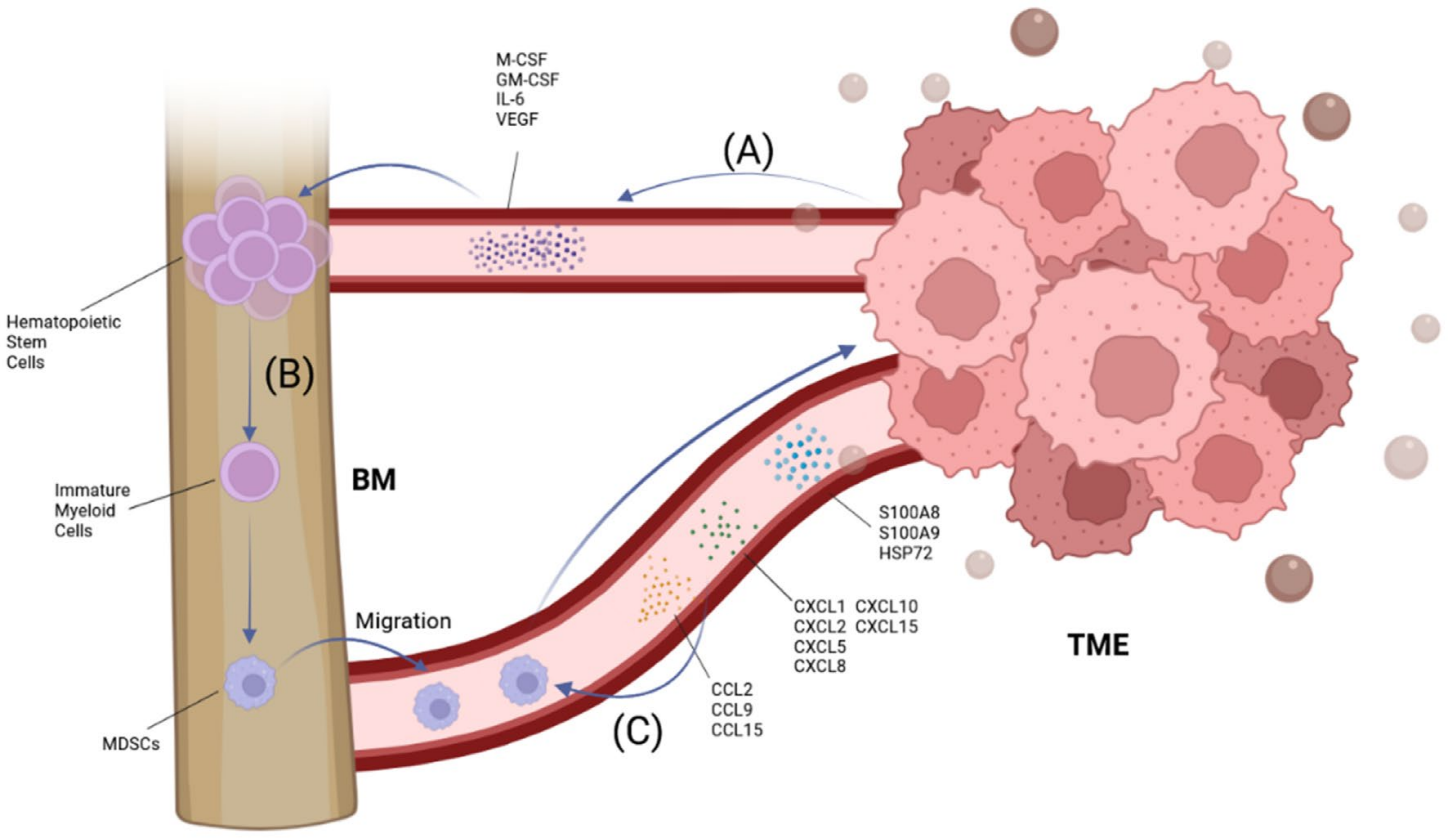
Tumor cells promote the migration of MDSCs to the TME. (A) In the TME, the primary tumor cells release variable kinds of molecules and exosomes inducing the recruitment of MDSCs through blood circulation. (B) In the bone marrow, tumor‐derived factors, such as IL‐6, M‐CSF, and GM‐CSF, direct the differentiation of hematopoietic stem cells to immature myeloid cells, including PMN‐MDSCs and M‐MDSCs. (C) Cytokines from tumor cells, such as CCL2, CCL15, HSP72, S100A8/S100A9, CXCL1, and CXCL2 guide the migration of MDSCs to the TME, enhancing the formation of immunosuppression environment in the TME.

In the past decades, a colossal number of teams focused on the mechanisms of MDSCs recruitment to TME for its crucial role in the related treatment. Based on the studies, we can conclude that the mechanisms include chemokines (Table 1), hypoxia circumstance, and exosomes. In this part of the review, we summarized some main axes of MDSCs recruitment (Figure 1), moreover, we focused on their relationships with bone metastatic cancer.

**TABLE 1:** Molecules associated with MDSCs recruitment to TME.

Molecule	Receptor	Model	Sites	References
CCL2/MCP‐1	CCR2	Colorectal cancer	Colon	[29]
CCL9	CCR1	Oral squamous cell carcinoma	Tongue	[30]
CXCL1	CXCR2	Colorectal cancer	Liver	[31]
CXCL2	CXCR2	Bladder cancer	Bladder	[32]
CXCL5	CXCR2	Breast cancer	Bone	[33]
CXCL8/IL‐8	CXCR2	Prostate cancer	Prostate	[34]
CXCL10	CXCR3	Melanoma	Lung	[35]
CXCL15	CXCR2	Prostate cancer	Prostate	[36]
KAT6A	SMAD3	Breast cancer	Lung	[37]
HSP72	TLR2	Colon carcinoma	Colon	[38]
S100A8/A9	TLR4	Breast cancer	Lung	[39]
VEGF	VEGFR	Ovarian cancer	Ovarian	[40]
PDL1	PD1	Hepatocellular cancer	Liver	[41]
POSTS	—	Breast cancer	Lung	[45]
LOXL2	—	Hepatocellular cancer	Lung	[42]
NOTCH	—	Glioma	Brain	[43]
OLFM4	—	Colorectal cancer	Colon	[44]

### 2.1 C‐C Motif Chemokine Ligand

In previous studies, C‐C motif chemokine ligands (CCL) serve as major components of the TME, impacting the progression of tumors. Some of them can restrict tumor expansion through accumulating inflammatory cells. However, some can also promote tumor growth via recruiting immunosuppression cells, such as MDSCs, especially CCL2. In oral squamous cell carcinoma (OSCC), CCL2 expression levels were markedly high in the patient‐derived stroma, and it was the key factor in recruiting MDSCs to the TME [46]. Moreover, multicolor immunohistochemistry (IHC) demonstrated that the migration of CD11b^+^ cells was suppressed via the absence of CCL2. Another pancreatic ductal adenocarcinoma (PDAC) research also verified that CCL2 enhanced the chemotaxis of M‐MDSCs [47]. In addition, the flow cytometry assays demonstrated that anti‐CCL2 antibodies effectively restrained MDSCs recruitment in PDAC tissues. Besides CCL2, other members also participate in the accumulation of MDSCs, for example, CCL5, CCL7, and CCL26. Some studies indicated that CCL5 can activate hypoxia‐inducible factor (HIF)‐1α and VEGF, which are critical in MDSC generation [48]. Further, there is a positive relationship between CCR5 expression and MDSC frequencies. In another study on uterine serous carcinoma, Mise et al. found that the CCL7 signal induced the migration of MDSCs [49], and MDSCs decreased after the suppression of CCL7 expression. Additionally, CCL26 has also been depicted as a pathway to recruit CX3C receptor‐1 expressing MDSCs. The research demonstrated that CCL26 was induced by hypoxia since it was a direct transcriptional target of hypoxia‐inducible factors (HIF) [50]. Through the formation of a hypoxia microenvironment, CCL2 induced the migration of MDSCs to the TME.

As for the relationship between CCL and bone metastasis, CCL2 plays an important role in the progression. Some previous studies suggested that CCL2 stimulated the bone metastasis of prostate and breast cancer in vivo [11, 51, 52, 53, 54]. A prostate tumor model designed by Mizutani et al. showed that there was a positive correlation between CCL2 expression and bone metastasis [11]. In the model, the X‐ray results demonstrated that CCL2 overexpression enhanced osteolytic bone metastasis, further, Mizutani et al. found that significantly more osteoclasts were observed in metastasis sites, which played an important role in the bone metastases. In addition, a recent report demonstrated that growth differentiation factor‐15 (GDF‐15) promoted the expression of CCL2 in osteoblasts [54]. In the study, the team examined whether GDF15 promoted bone metastasis in prostate cancer via tissue microarray. Moreover, further experiments suggested that this progression depended on CCL2 for its function in tumor‐associated macrophages. The inhibition of CCL2 significantly suppressed the osteoclast genesis directed by GDF‐15. Taken together, these data showed that CCL2 induced various mechanisms in bone metastasis of different cancers.

### 2.2 C‐X‐C Motif Chemokine Ligand

Like CCL, the C‐X‐C motif chemokine ligand (CXCL) also participates in the recruitment of MDSCs. Previous studies have discovered several members of the CXCL family promote the formation of the TME by inducing the accumulation of MDSCs. Among them, quite a lot of research has verified that CXCL1/2 can upregulate the population of MDSCs and induce poor clinical prognosis [31, 32, 55, 56]. For instance, in colorectal cancer (CRC) liver and lung metastases, recruitment of MDSCs into the premetastatic niche induced by CXCR2 and its ligands makes a great contribution, moreover, the CXCL1 levels are associated with the accumulation of MDSCs, tumor size, and invasive depth [31]. Besides CXCL1/2, other ligands also participate in the recruitment of MDSCs. In a recent study, Cao et al. examined the roles of CXCL5 in this progression [57]. They validated that chronic restraint stress‐induced hepatocellular carcinoma (HCC) progression. Moreover, through immunohistochemical staining analysis, they found the relation between HCC progression and the gathering of MDSCs. Subsequent results demonstrated that the interaction between CXCR2 and CXCL5 activated the mitogen‐activated protein kinase (MAPK) pathway to cause an impact on the recruitment and survival of MDSCs. In the latest study, MDSCs can be recruited into the TME in response to CXCL16, moreover, the expression of S100A9 stimulates the accumulation of cancer‐associated fibroblasts (CAFs) [58]. The activated CAFs in turn produce more CXCL16 to increase the population of MDSCs. Besides these, Sun et al. demonstrated that CXCL10–CXCR3 also contributed to promoting MDSCs migration [59]. In the study, Sun et al. indicated that M‐MDSCs can be accumulated and activated by the p38 MAPK signaling pathway through CXCR3. Taken together, tons of studies have verified the relationship between CXCL and MDSCs, all these pathways will provide novel immune treatments in the future. For example, related cytokine inhibitors can be applied to regulate the infiltration of MDSCs and suppress tumor progression.

CXCL also participates in cancer‐related bone destruction for its role in bone metastasis [33, 60, 61]. For instance, Oue et al. found that CXCL2 expressed by oral squamous cell carcinoma enhanced the increase of osteoclasts and induced bone metastasis. Moreover, it can be dose‐dependently inhibited by relevant antibodies [62]. Besides, CXCL5 also plays a role in the bone metastasis of prostate cancer [33]. The concentration of CXCL5 is positively correlated to the proliferation of the cancer cells, further, it prompts bone colonization. Additionally, preventing the interaction between CXCR2 and CXCL5 can attenuate the metastasis progression. Combining the effects of CXCR1/2 on MDSCs recruitment and bone metastasis, it is obvious that MDSCs play a crucial role in bone metastasis via the CXCR1/2 axis, moreover, it can be a possible target for clinical trials.

### 2.3 Lysine Acetyltransferase 6A (KAT6A)

Lysine acetyltransferases (KATs) consist of three families related to the amino acid sequence, the MYST family, the p300/CBP family, and the Gcn5‐related family. KAT6A is a member of the MYST family, contributing to enhancing the acetylation of lysine residues [63]. Previous studies have indicated that KAT6A proteins augment the progression and expansion of several kinds of tumors [64, 65, 66]. Novel research reveals that KAT6A also participates in the regulation of MDSCs recruitment. In the triple‐negative breast cancer model designed by Yu et al., the KAT6A‐SMAD3 axis resulted in cancer immune escape and metastasis [37]. SMAD2 and 3 belong to the family of transcription factors, which is the core of the transforming growth factor‐β (TGF‐β) pathway. The MS analysis validated that the interaction between KAT6A and SMAD3 is the acetylation of lysine residues at lysine 20 and 117. The further experiment indicated that the acetylation of SMAD3 prompted the recruitment of MDSCs to the metastatic sites. Moreover, profound validation suggested that the KAT6A knockdown decreased metastasis in the lungs and MDSCs accumulation in the TME [37]. In addition, KAT6A can also up‐regulated inflammatory cytokines such as IL‐6, which can significantly enhance the differentiation and accumulation of MDSCs in the TME via IL‐6/STAT3 axis.

Additionally, the KAT6A‐SMAD3 axis can also affect the bone metastasis of tumors. In previous research carried on by Petersen et al., SMAD2 and SMAD3 played opposing roles in breast cancer bone metastasis [67]. Their assessment depended on silencing SMAD2 and SMAD3. The result demonstrated that SMAD2‐deficient cells showed a more aggressive phenotype than the other groups, while SMAD3‐deficient cells presented a mild phenotype in the bone metastatic sites. Further, the fluorescence imaging demonstrated that the SMAD2‐silenced mouse suffered more serious breast cancer bone metastasis in the femur, tibia, and vertebra, however, the SMAD3‐silenced mouse presented a contrast result. Combining these two experiments, we can conclude that the AKT6A‐SMAD3 axis plays a crucial role in bone metastasis, further, it can also augment the accumulation of MDSCs in the bone metastasis niches.

Another possible pathway is the interaction between KAT6A and PI3K/AKT. PI3K/AKT has been proven that this signaling pathway can increase the production of CXCL1 and other chemokines, further, some studies demonstrated that deficient SLC7A2 augmented the progression of tumors and the recruitment of MDSCs through this pathway [68, 69]. Lv et al. have validated that KAT6A can upregulate the PI3K/AKT signaling pathway in a previous study [70]. They found that KAT6A enhanced PIK3CA expression in the gliomas model, further, it could activate PI3K/AKT signaling. The team assessed the influence of KAT6A energy on the expression of PIK3CA in glioma cells, and the result indicated that it effectively inhibited the expression of PIK3CA and the activation of the PIEK/AKT signaling pathway. These experiments suggest that KAT6A can recruit MDSCs through the activation of the PISK/AKT pathway.

### 2.4 Hypoxia and Hypoxia‐Inducible Factor‐1

Hypoxia is a crucial environmental feature of premetastatic niche and TME, as the oxygen consumption of tumor cells exceeds the supply of functional blood vessels [71]. The major molecular mechanism of hypoxia is associated with the hypoxia‐inducible factors (HIFs) [72], which orchestrate the immune environment in the premetastatic niche by inducing numerous chemokines such as C‐C motif chemokine ligand 26 (CCL26) [73]. Additionally, hypoxia‐inducible factor‐1 (HIF‐1) also stimulates the nuclear factor kB (NF‐kB)‐granulocyte colony‐stimulating factor (G‐CSF) axis and promotes G‐CSF production [74]. It has been reported by Kawano et al. that G‐CSF can induce the accumulation and prolong survival of MDSCs through the JAK/STAT3 signaling pathway [75]. Apart from these, extracellular Adenosine triphosphate (ATP) hydrolyzation to 5′‐AMP affected through ectonucleoside triphosphate diphosphate hydrolase (ENTPD), an important transcriptional target of HIF‐1, and promotes the accumulation of MDSCs in hepatocellular carcinoma (HCC) model [73]. ENTPD anergy in HCC cells down‐regulated the accumulation of MDSCs suggesting that ENTPD plays a key role in the accumulation of MDSCs, and the expression of 5′‐AMP positively related to the accumulation of CD11b^+^ myeloid‐derived cells. In a recent study, the HIF inhibitor 32‐134D gained the expected treatment prognosis in combination with anti‐PD1 immunotherapy since it led to a reduced population of TAMs and M‐MDSCs by inhibiting the CXCL1, IL‐6, and IL‐10 [76].

As a common form of metastasis, bone metastasis is also regulated by hypoxia and HIF. In a mice model designed by Devignes [77], HIF signaling in osteoblast prompted the bone metastasis of breast cancer. Moreover, this signaling increases the growth of breast cancer. Another review indicates that HIF signaling has an impact on the epithelial–mesenchymal transition (EMT) directly and indirectly [78]. In addition, hypoxia can also play an important role in prompting osteolysis and preparing for colonization [79].

### 2.5 Exosomes

Exosomes are a kind of endocytic membrane vesicle secreted by different types of cells, especially the tumor cells, which prompt the metastatic sites formation [80, 81, 82, 83]. Tumor‐derived exosomes (TDEs) perform several biology functions, particularly cellular communication induced by delivering proteins, RNAs, and DNA segments [84, 85]. It has been reported in previous studies that TDEs play a crucial role in premetastatic niche formation, especially in the recruitment of immune suppression cells, such as immature myeloid cells expressing CD11b^+^ and Tregs [80, 86]. Wang et al. have reported that the stimulation of inflammation molecules prompted by TDEs, such as HSP72, PGE2, S100A8/A9, interleukin (involving IL‐10, IL‐6), and vascular endothelial growth factor (VEGF), can affect myeloid cells through facilitating their migration to premetastatic niche sites [84]. The relationship between MDSCs and MMPs has also been identified by previous studies [87, 88]. Zhang et al. used a lung cancer model and demonstrated that an increase in MMP activation led to the accumulation of M‐MDSCs [87]. Another study showed that exosomes can enhance the expression of IL‐6 in the monocytes, which then upregulates the JAK/STAT3 signaling pathway and accumulates MDSCs in premetastatic sites [89]. Generally, the proinflammation with related molecules is considered the critical mechanism of exosome‐induced MDSCs recruitment.

Besides the mechanisms depending on inflammation molecules, exosomes mediate the migration of MDSCs to the TME through Phosphatase and tensin homolog (PTEN) silencing [74]. The loss of PTEN expression increases the secretion of the chemokine CCL2 which can recruit myeloid cells to induce the immune repression in premetastatic niches. Another TDE‐related mechanism is glycolytic dominant metabolic reprogramming proposed by Morrissey et al. [90], which illustrated that macrophages within the premetastatic niche acquire the immunosuppression phenotype in this novel way and identified the link with exosomes. Morrissey et al. discovered that the TDE‐induced upregulation of the NF‐kB/PD‐L1 pathway was stimulated by metabolic reprogramming, and exosomes and PD‐L1 promoted the accumulation and immunosuppression phenotype polarization of M2 macrophages [91, 92]. Furthermore, the TDE‐augmented macrophages demonstrated a dominant rise in the releasing of IL‐10, IL‐6, G‐CSF, and CXCL1, which can further recruit MDSCs to the microenvironment [90]. Taken together, exosomes are crucial to the recruitment and differentiation of MDSCs, thus, TDEs present great potential in the immunotherapy field.

Several articles have reported the role of exosomes in prostate and breast cancer bone metastasis [93, 94, 95, 96, 97, 98]. Akoto and Saini demonstrated that exosomes mediated communication in premetastatic niche formation, including accumulating fibronectin and promoting ECM‐remodeling [98]. In addition, exosomes up‐regulated osteoblast activity, promoting the establishment of bone metastasis. Generally, it is necessary to do some further research on the relationship among MDSCs, TDEs, and tumor bone metastasis to guide the potential clinical treatment.

### 2.6 Periostin

The role of periostin (POSTN) in regulating the immunosuppressive environment in tumor metastasis sites was seldom documented. Previous studies have proved that POSTN participated in the formation of premetastatic niches [99]. Wang et al. demonstrated the correlation between POSTN and MDSCs in breast tumor pulmonary metastasis [45]. First, POSTN was positively correlated with MDSCs as POSTN deficiency decreases CD11b^+^ cells in mice. Furthermore, the metastatic sites exhibited high expression in S100A8 and LOX, which are associated with the migration of MDSCs. In addition, this study found that POSTN promoted LOX activation in the breast tumor model and the deficiency of POSTN decreased the immunosuppression functions related to activating ERK and AKT in MDSCs and impaired the MDSCs‐induced tumor metastases. In another study, the results demonstrated a positive relationship between POSTN and CCL2 expression [100], which suggests that POSTN can regulate the recruitment of MDSCs through the CCL2 axis. Taken together, POSTN promotes the gathering of MDSCs in tumor metastases, moreover, its deficiency represses the immunosuppressive function of MDSCs.

Previous research proved that POSTN played crucial roles in bone metastasis and osteosarcoma [101, 102, 103, 104]. An osteosarcoma model designed by Alfino et al. indicated that low expression of POSTN heightened immunity [102], moreover, in periostin overexpressing osteosarcoma, EMT was significantly enriched, and it was related to poor clinical prognosis. Another report showed the role of POSTN in breast cancer metastasis [103]. The antibodies of POSTN inhibit tumor proliferation and induce tumor necrosis, further, it can suppress the migration to the bone.

### 2.7 Toll‐Like Receptors

Toll‐like receptors (TLRs) are members of pattern recognition receptors, as a crucial way to stimulate innate immune reactions against infections. In recent studies, it has been verified that TLRs participate in anti‐tumor reactions by stimulating the activation of T cells, NK cells, and dendritic cells [105, 106]. However, the relationship between TLRs and MDSCs is still unclear, latest studies suggest that TLRs seem to play a dual role in the accumulation and tumor‐promoting effect of MDSCs, depending on the subtypes of TLRs [107, 108, 109].

A recent study verified that TLR‐7 promotes tumor progression through the recruitment of MDSCs [107]. The researchers discovered that CL264, the agonist of TLR‐7 induced a pro‐tumorigenic effect. Furthermore, the results demonstrated that the frequencies of MDSCs in TLR‐7 KO mice were significantly lower than those in WT mice. Moreover, the study suggested that the pro‐tumorigenic effect of MDSCs was positively associated with TLR‐7 stimulation. Another research showed that TLR‐2 played a similar role in the recruitment of MDSCs [109]. According to the study result, it suggests that the activation of TLR‐2 induces the increase of MDSCs. Moreover, Shime et al. verified that TLR2 enhanced the immunosuppression of M‐MDSCs via Nitric oxide synthase (NOS). TLR‐9 is another member of TLRs which is related to the MDSCs. In a recent study, the TLR‐9 was up‐regulated in pancreatic ductal adenocarcinoma (PDAC) [110]. Researchers depicted that TLR‐9 accelerated the progression of PDAC and stimulated the reprogramming of pancreatic stellate cells (PSCs). Further research demonstrated that TLR‐9 activation sustained a proinflammatory microenvironment, and the data also suggested that MDSCs may be recruited to TME via TLR‐9.

Although there are various studies suggesting that TLRs play a significant role in tumor progression and MDSC recruitment, it is still unclear whether they promote bone metastasis. However, some research on TLRs may provide indirect evidence. For instance, a previous study demonstrated that TLR‐9 enhanced prostate cancer progression by augmenting the immunosuppressive effect of PMN‐MDSCs [111]. Since bone metastasis is one of the most significant pathways in prostate cancer, we hypothesize that TLR‐9 may promote the bone metastasis of prostate cancer via stimulating MDSCs, and it depends on further studies to verify.

## 3 Immunosuppression Mechanism of MDSCs in TME

**FIGURE 2:**
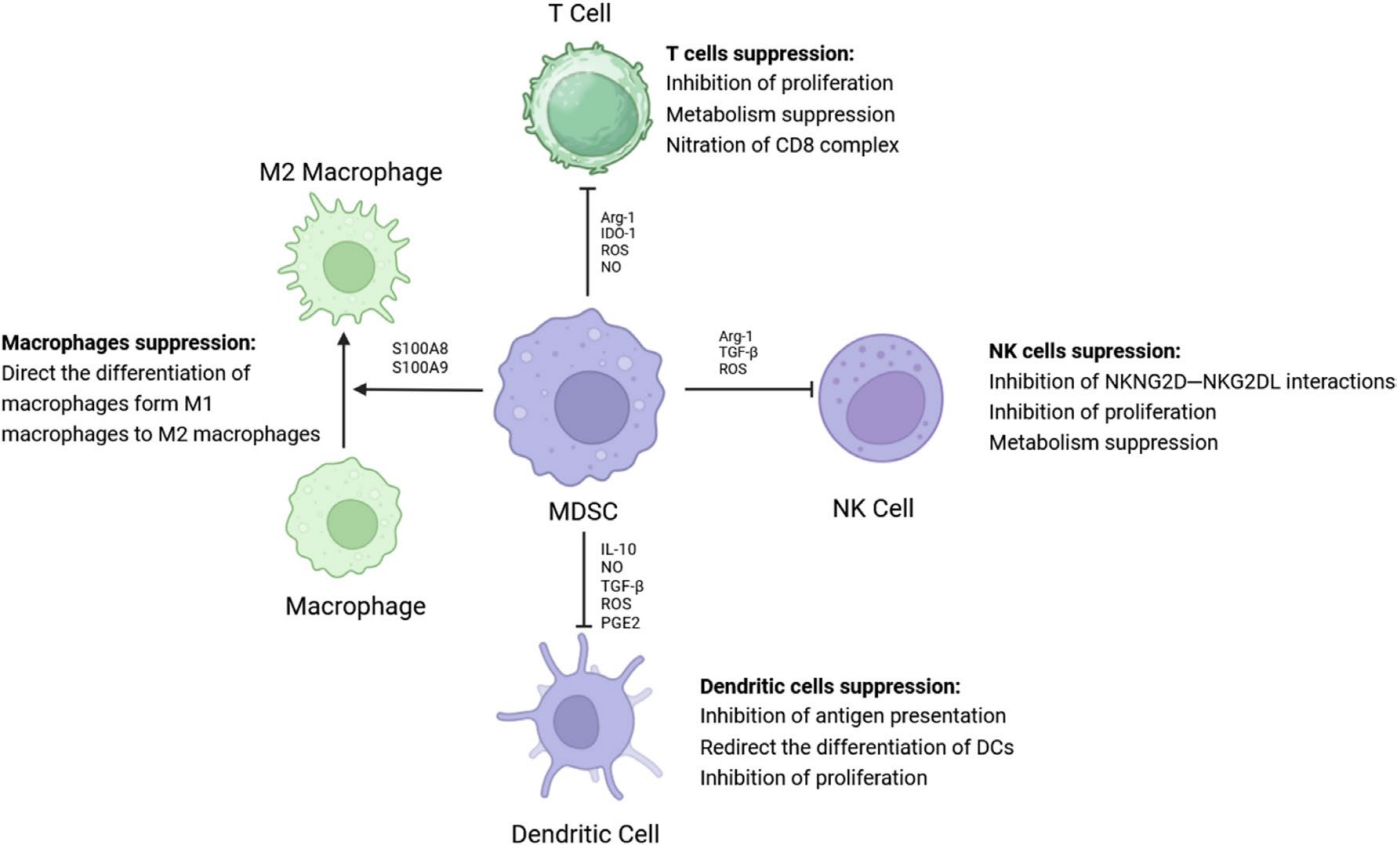
The immunosuppression mechanisms induced by MDSCs. (A) The main interaction between MDSCs and T cells is mediated by Arginase I (Arg1), ROS, IDO‐1, and NO. Most are related to the metabolism of amino acids, for example, arginine and cystine. (B) The immune suppression of NK cells is induced by the inhibition of NKG2D–NKG2DL interactions, which is influenced by factors such as TGF‐β. Other mechanisms are also correlated with the metabolism of amino acids, such as Arg 1 and ROS. (C) Dendritic cell inhibition is caused by the interaction of IL‐10, TGF‐β, NO, and ROS. (D) MDSCs can guide the differentiation to M2 macrophages through S100A8/S100A9, which are also called TAMs. The TAMs present the immunosuppression abilities.

Since the discovery of MDSCs, it has been widely accepted that MDSCs play a key role in immunity dysfunction and tumor expansion in the TME via various mechanisms (Figure 2). Moreover, these functions depend on the suppression of immune cells, including T cells, natural killer cells (NK cells), and macrophages. Here, we address some classical and innovative mechanisms of immune suppression in TME.

### 3.1 T Cells

T cells are basic participants of adaptive immunity, majorly including CD4+ and CD8+ T cells. In previous research, the suppression of T cells always played a critical and vital role in the study of the immunosuppression mechanisms of MDSCs. Generally, it includes amino acids metabolism disturbance, production of reactive oxygen species (ROS), and nitric oxide synthase (NOS) [13, 112].

#### 3.1.1 Amino Acids Metabolism Disturbance

In previous studies, disturbing T cells' amino acid metabolism is always a key role in the immunosuppression of MDSCs. It is well known that MDSCs highly express arginase 1 in the TME [113], and it causes the depletion of L‐arginine [114], which is needed for the proliferation of T cells. L‐arginine starvation results in the silencing of cyclin D3 and CDK4 [115], and T cells are arrested in the G0–G1 phase of the cell cycle [116], suggesting that the absence of L‐arginine repressed the proliferation of T cells and diminish the immune response. Arginase 1 also affects the energy metabolic pathways of T cells. A previous study showed that there was an obvious decrease in glucose uptake and glycolysis when T cells were treated with arginase 1 [116]. Indoleamine 2,3‐dioxygenase (IDO) is another key mechanism associated with tryptophan catabolism [117]. IDO participates in the immune evasion induced by tumor‐infiltrating MDSCs [118, 119], and one of the most important roles of IDO is activating regulatory T cells via catabolizing tryptophan and decreasing the local concentration of free tryptophan. In the breast cancer model, Yu et al. found that IDO expression in MDSCs was positively correlated with the infiltration of Tregs [120]. Furthermore, the accumulation of catabolites such as kynurenine reduces the survival of CD4^+^ T cells [117, 121]. Interestingly, combining IDO‐1 and checkpoint inhibitors was confirmed to be significantly comparable with monotherapies in previous studies [118, 122], which demonstrated the possibility of this therapeutic strategy. Cysteine starvation is also associated with the anergy of T cells [113, 123, 124]. Srivastava et al. described that MDSCs competitively imported cysteine, but they did not export cysteine as other antigen‐presenting cells, due to the absence of transporters [119, 125]. Therefore, T cells cannot obtain adequate cystine for proliferation and activation. In addition, the immune suppression can be partially reversed by providing extracellular cysteine [125].

#### 3.1.2 Production of ROS, NO, and NOS

ROS in MDSCs include superoxide and hydrogen peroxide, and hydrogen peroxide is the major component [126, 127, 128]. Former research has indicated the role of ROS in tumor‐infiltrating MDSCs, and MDSCs express elevated levels of ROS in different cancer models. Cells can produce ROS via numerous mechanisms, but the major source of ROS in MDSCs is the activity of the family of NADPH oxidases (NOXs) [129, 130, 131]. NOX is an enzyme involving two membrane proteins and four cytosolic components. Corzo et al. found that the activity of NOXs was regulated by STAT3 transcription factors [129], and selective STAT3 inhibitor JSI‐124 caused a dramatic decrease in the expression of NOXs and reduced the levels of ROS in MDSCs. ROS plays an important role in tumor growth, progression, and metastasis. As signaling molecules, ROS drive the EMT [132, 133], which is a crucial step in tumor metastasis. Interestingly, although MDSCs express high levels of ROS, they are not affected by oxidation stress since they also express high levels of NRF2 [134], which is a master regulator of intracellular antioxidant responses. ROS also plays an important role in osteosarcoma progression and tumor bone metastasis [135, 136, 137, 138, 139]. A recent report demonstrated that Meso‐Hannokinol (HA) could inhibit breast cancer bone metastasis via the ROS/JNK axis [135]. Zhu et al. found that HA effectively down‐regulated in vivo and in vitro migration of breast cancers. Furthermore, HA inhibited EMT and osteoblast activators in breast cancer cells, including MMP‐9 and MMP‐13, which regulate breast cancer‐induced osteolysis.

Nitric oxide synthase (NOS) has three isoforms, including neuronal NOS (nNOS), endothelial NOS (eNOS), and inducible NOS (iNOS) [140]. Indeed, iNOS is the main participant in the immune suppressive function of MDSCs [141] and induces a poor prognosis in cancer diseases, whereas the other two isoforms make contributions to healthy tissues [140]. Moreover, the iNOS‐selective inhibitor can reverse immune suppression and increase the expression of M1 macrophages [142]. As the substrate of iNOS in MDSCs, L‐arginine is catabolized into nitric oxide (NO) and L‐citrulline [57, 143, 144]. Accumulating NO is positively correlated with the expression of COX‐2 and HIF‐1a [145], both of which promote the recruitment and survival of MDSCs.

The key mechanism induced by ROS and iNOS is the interaction of NO and superoxide, which produces a high level of peroxynitrite with a high biological activity [114, 142, 146]. Additionally, the peroxynitrite induces the nitration of the T cell receptor‐CD8 complex [56, 112, 147], and it further damages the binding to the peptide MHC class I complex, which results in non‐response to antigen‐specific stimulation. In addition, a previous report indicated that reactive nitric species could modify the chemokines such as CCL2 [148], inhibiting the induction of anti‐tumor lymphocytes. Interestingly, this mechanism does not influence MDSCs recruitment, which is partially regulated by CCL2.

The inhibition of T cells in the metastatic sites plays a significant role in the progression of bone metastasis, and some studies have indicated this phenomenon [149, 150, 151, 152, 153]. For instance, Arellano et al. found that in the metastatic niches of breast cancer cells, T cells enhanced osteolytic bone metastases and prompted the osteolysis area [149]. Surprisingly, when Arellano et al. added the T cells from healthy mice, it did not show the same result, instead, the T cells inhibited the progression of tumors. Profound experiments demonstrated that mature T cells such as Th1, Th2, and cytotoxic T cells were decreased in bone metastases. This result indicated that immature T cells induced the progression of bone metastases. To verify the mechanism of immunosuppression in bone metastatic sites, the team used cytometry analysis, and they observed that there was an increase of MDSCs in bone metastases of breast and prostate cancer. Moreover, further co‐culture examination suggested that these MDSCs did play a crucial role in bone metastases and T cell suppression. Collectively, we anticipate further study will show us more pathway to inhibit tumor bone metastasis by regulating amino acid metabolism.

### 3.2 NK Cells

As another important component of the immune system, NK cells play a crucial role in immunosurveillance. Some studies reported that MDSCs harm the immune functions of NK cells [72, 154, 155, 156, 157, 158, 159, 160, 161]. In a mice model designed by Moshe Elkabets et al., Ly6C^−^ MDSCs inhibited NK cell immune functions in vivo [154]. In the study, Moshe et al. observed that there was an exponential reduction of NKG2D expression by NK cells when co‐cultured with MDSCs. NKG2D is a special receptor expressed in NK cells and T cells, previous research demonstrated that the high‐affinity NKG2D ligand released by tumor cells can stimulate NK cells and enhance their antitumor functions [162]. Additionally, in a melanoma model, it is observed that the inhibition of MDSCs expansion restores the infiltration and immune functions of NK cells. In this study, Isak W Tengesdal et al. found that the inhibition of tumor‐derived NLRP3 by OLT1177 reduces MDSCs expansion, further, it results in reduced tumor size with increased infiltration of NK cells [159].

TGF‐β plays another critical role in the suppression of NK cells [156]. TGF‐β is one of the major immunosuppression mechanisms, and previous research reported the correlation between MDSCs and TGF‐β [163]. In the study, treatment with 1D11, the inhibitor of TGF‐β, significantly decreased the expression of Arginase 1. In addition, the neutralization of TGF‐β reduced the number of MDSCs in TME, further decreasing the size of the tumor. Another study designed by Li et al. indicated that MDSCs induced the anergy of NK cells through membrane‐bound TGF‐β [163]. In the study, Li et al. discovered that in the Transwell system, MDSCs downregulated the expression of NKG2D, suggesting that MDSCs played a crucial role in inhibiting the immune function of NK cells. Further, the team examined the influence of TGF‐β in this process, and the obvious result that neutralization of TGF‐β in the co‐culture system did restore NKG2D expression proved the idea. In another osteosarcoma model, the team found that osteosarcoma cells highly expressed NKG2D ligands, which could direct NKG2D–NKG2DL interactions [164]. Via this interaction, NK cells showed the ability to kill osteosarcoma cells. Further, blocking the NKG2DLs induced a significant decrease in NK cells' cytotoxicity against osteosarcoma. Taken together, MDSCs also impose a negative effect on the interaction between NK cells and osteosarcoma cells.

As a major component of the immune system, the roles of NK cells in bone metastasis have been summarized in previous studies [165, 166, 167]. Generally, NK cell dysfunction has been observed in the metastatic sites [168], which indicates that MDSCs‐induced anergy of NK cells also plays an important role in the formation of metastatic niches. Furthermore, following this pathway, some trials have been carried out exploring the possible treatment targets [169, 170, 171, 172]. For example, in a recent report, Wu et al. found that SCUBE2 mediated bone metastasis of breast cancer cells by promoting osteoblast differentiation and NK cells dysfunction [172]. The article pointed out that the SCUBE2 could promote osteoblasts differentiation, moreover, these osteoblasts inhibited the NK cells via downregulating the collagen‐LAIR1 signaling pathway. Another novel study of Pal et al. demonstrated that the microbiome suppressed melanoma bone metastasis through promoting NK cells and Th1 cells homing to bone. In this study, researchers found that antibiotics‐treated mice showed much more severe melanoma bone metastasis. Further experiments verified that the depletion of microbiome hurt the infiltration and function of immune cells, especially NK cells. These studies further confirmed the importance of NK cells in inhibiting bone metastasis, suggesting that the interaction between MDSCs and NK cells may enhance the bone metastasis.

### 3.3 Dendritic Cells

Dendritic cells (DCs) are a group of professional antigens presenting cells, and they play a crucial role in anti‐infection and anti‐tumor functions. In TME, MDSCs inhibit the immune efficiency of DCs in different kinds of ways, involving IL‐10, NO, and TGF‐β [173, 174, 175, 176, 177, 178]. In a previous study, Cheng et al. demonstrated that S100A9 protein could regulate the accumulation of MDSCs and the inhibition of DC differentiation [179]. In the report, Cheng et al. indicated that S100A9 upregulated the production of ROS in MDSCs, further, the substantially increased ROS level inhibited DC differentiation. Another recent study demonstrated that NO produced by MDSCs can inhibit DC immune responses in cancer [180]. The team discovered that NO prevented antigen presentation from DC to CD4^+^ T cells. In addition, adding the inhibitor of iNOS, L‐NAME, successfully restored the immune functions of DCs, suggesting that MDSCs can inhibit DCs through the production of NO. PGE2 is another mechanism that MDSCs inhibit DC immune functions, furthermore, PGE2 and COX2 can redirect the differentiation of DCs toward MDSCs. In the study designed by Obermajer et al. [181], PGE2/COX2 mediated positive feedback loop induced the production of MDSCs‐associated suppressive factors, involving PD‐L1, IDO‐1. Furthermore, this positive loop redirected the GM‐CSF and IL‐4‐driven differentiation of DCs to MDSCs. In another HCC mice model, it was found that the number of DCs was decreased by MDSCs via IL‐10. The report showed that the interaction between DC and MDSCs resulted in a decrease in the production of IL‐12 and T‐cell stimulatory activity. Furthermore, the team measured the supernatant cytokines in the co‐cultured system, the result suggested that IL‐10 may induced the down‐regulated IL‐12 production from DCs.

Some studies also found that DC also has immunosuppressive effects in metastatic niches, which are mainly induced by plasmacytoid dendritic cells (pDC). In addition, the network between pDC and MDSCs can boost the metastasis progression of cancer cells, such as bone metastasis [182]. Previous reports have indicated that MDSCs in bone microenvironment could differentiate into osteoclasts, which can prompt bone metastasis and osteolytic lesions. Sawant and Ponnazhagan indicated that the differentiation was induced by pDC, and the results suggested that it was related to the IL‐10 secreted by Th2 cells [183]. In addition, the combination treatment has been proven effective in depleting both pDC and MDSCs. On top of IL‐10, the RANKL–RANK axis in the bone microenvironment also plays a crucial role in the interaction between pDC and MDSCs, assisting the differentiation from MDSCs to osteoclasts. The studies have indicated that this axis contributed to bone metastatic tumor growth and progression [184]. Generally, these studies suggest that depletion of both pDC and MDSCs, combining enhancing anti‐tumor effects of DC can become the future clinical research target.

### 3.4 Macrophages

In TME, macrophages are divided into two types, M1 and M2 [185]. The former one is classically activated macrophage, which presents anti‐tumor effects, including the direct killing of tumor cells, secreting various cytokines, and suppression of metastasis. The latter one is also called alternatively activated macrophage. M2 cells are the most representative cells of TAMs, with the effects of immunosuppression and promoting metastasis. The shift induced by MDSCs from M1 to M2 is the major mechanism that manipulates the function of macrophages [178, 186]. Furthermore, MDSCs also differentiate from the M2 macrophage phenotype in TME [14, 185, 187, 188]. Alexandra Pritchard et al. observed that in a lung cancer environment, the exosomes from cancer cells enhanced the differentiation of M2 macrophages from MDSCs [92]. As for M2 macrophages or TAMs, previous studies have demonstrated the negative immune effects in TME and poor clinical prognosis. For instance, in a report related to HCC, Zhang et al. indicated that the infiltration of TAMs enhanced angiogenesis and tumor metastasis, further, it caused worse overall survival and recurrence‐free survival [189]. In addition, TAMs also participate in the bone metastases of prostate cancer [190, 191]. Fabiana et al. verified that macrophage depletion in mice can repress prostate tumor growth in bone [190]. Further, macrophage ablation in bone metastatic niches also inhibits osteolysis, which plays a critical role in the progression of migration to bone. The following experiments also suggested that M2 macrophage depletion can suspend the subcutaneous growth of prostate cancer in an implant model. Taken together, MDSCs‐induced differentiation of M2 macrophages or TAMs augments the growth of tumors and migration to the bone, and the depletion of such kinds of macrophages can significantly inhibit the volume and growth of tumors.

Surprisingly, M1 macrophages can prompt the accumulation of MDSCs [192]. In a study designed by Tu et al., IL‐1β expressed by M1 macrophages recruited MDSCs to TME [193]. Furthermore, IL‐1 activated MDSCs through the NF‐κB signal pathway. Tu examined the mRNA expression of NF‐κB target genes, and it increased significantly in MDSCs isolated from IL‐1β transgenic mice. In addition, M1 macrophages can also enhance MDSC accumulation through TNF‐α [194].

Taken together, as the precursor of TAMs or M2 macrophages, MDSCs can enhance the differentiation of M2 macrophages, moreover, macrophages can also prompt the accumulation of MDSCs through IL‐1β, TNF‐α, and other cytokines.

**FIGURE 3:**
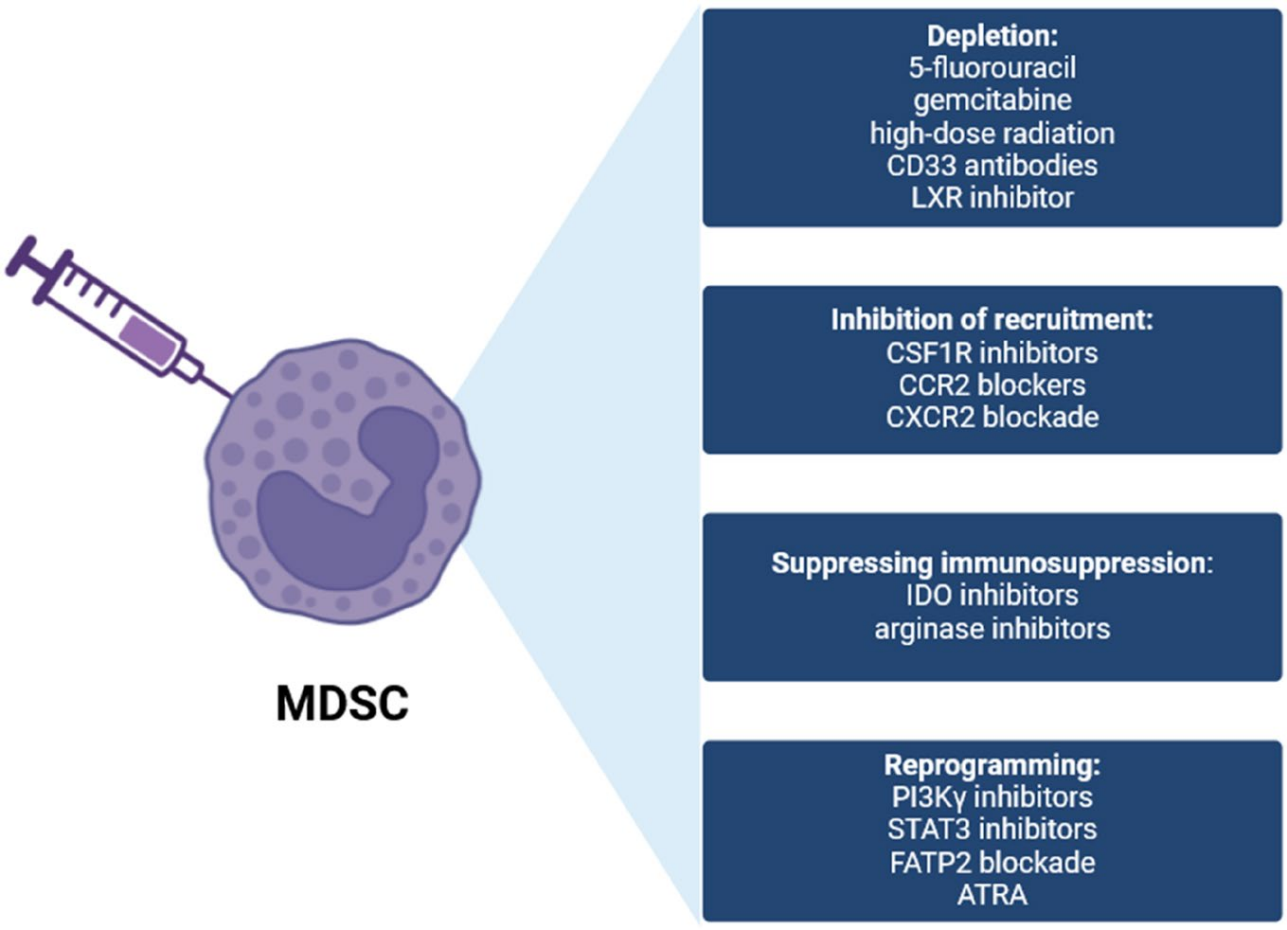
Targeting MDSCs in cancer therapy. (A) Depletion of MDSCs: The direct way to avoid the intervention to ICB is depleting MDSCs. Low‐dose chemotherapeutic drugs, high‐dose radiation therapy, CD33 antibodies, and liver X receptor (LXR) inhibitors have been proven to achieve this in previous studies. (B) Targeting MDSC recruitment: According to the mechanisms of MDSCs recruitment, it is obvious that blocking these pathways will inhibit MDSCs migration. Previous studies have tried following molecules: CSF1R inhibitors, CCR2 blockers, and CXCR2 blockade. Some have been tried in pre‐clinical trials and showed a satisfying result. (C) Targeting MDSC immunosuppression function: The suppressive immune functions of MDSCs are related to immune cells' metabolism. Based on this, some targeting immunometabolism pathways have been tried, such as arginase inhibitors and IDO inhibitors. However, previous studies found that metabolic adaptation decreased the efficiency of targeting individual metabolism. (D) Reprogramming MDSCs: The differentiation of MDSCs can be regulated by small molecules associated with related axis, such as PI3Kγ inhibitors, STAT3 inhibitors, C/EBPα, FATP2 blockade and all‐trans retinoic acid (ATRA). Reprogramming MDSCs can activate their anti‐tumor response, improving ICB efficiency.

Combining the mechanisms of MDSCs recruitment and immunosuppression, several possible treatment methods have been applied to inhibit MDSCs (Figure 3). The first way is the blockade of MDSC recruitment. Considering the critical role of the STAT3 pathway in MDSCs accumulation, suppressing STAT3 can be a feasible way to block MDSCs accumulation, and a relevant study has verified the idea [195]. Another possible way is to restrict the immunosuppression function. As ROS is an essential mechanism suppressing immune function, it is a reasonable way to inhibit ROS. Li et al. discovered that all‐trans‐retinoic acid (ATRA) can alter MDSCs function via downregulation of ROS [29]. Further, combination therapy with ARTA and PD‐1 did present a better prognosis. Based on the profound research on MDSCs, we can infer that more available treatment methods will be put forward.

## 4 Conclusion

In this century, with the findings of immune checkpoint molecules, immunotherapy has gained remarkable advances. However, immune evasion significantly affects the efficacy of immunotherapies, and key factors are multiple mechanisms inducing immunosuppression. In this background, MDSCs have been regarded as the critical participants. Emerging research suggests that MDSCs play significant roles in the formation of the immune suppressive environment. Current studies have demonstrated many kinds of characteristics of MDSCs. However, there are still lots of problems to overcome. For example, although it has been proven that MDSCs present immunosuppression in TME, the situation is quite different in chronic inflammation and autoimmune diseases. Some studies have found that their accumulation is correlated with disease severity. Therefore, further research is needed to identify their roles in different environment. These questions can be answered by scRNA‐seq and other advanced quantitative technologies. Further and more profound studies will provide us more aspects to understand the essence of MDSCs. Additionally, in the field of bone metastasis, the role of MDSCs still needs to be identified, and profound research can show us more details that how MDSCs affect the progression of bone metastases. In the coming future, there will be more insights on these problems, and these findings will facilitate our understanding of the clinical use of these cells.

## Author Contributions

All authors contributed equally to all aspects of the article.

## Conflicts of Interest

The authors declare no conflicts of interest.

## Data Availability

The data that support the findings of this study are openly available in Figshare at https://doi.org/10.1002/cnr2.70044.
